# Resequencing *PNMT *in European hypertensive and normotensive individuals: no common susceptibilily variants for hypertension and purifying selection on intron 1

**DOI:** 10.1186/1471-2350-8-47

**Published:** 2007-07-23

**Authors:** Katrin Kepp, Peeter Juhanson, Viktor Kozich, Mai Ots, Margus Viigimaa, Maris Laan

**Affiliations:** 1 Department of Biotechnology, Institute of Molecular and Cell Biology, University of Tartu, Tartu, Estonia; 2 Institute of Inherited Metabolic Diseases, Charles University – First Faculty of Medicine, Prague, Czech Republic; 3 Department of Internal Medicine, University of Tartu, Tartu, Estonia; 4 Division of Cardiology, Northern Estonian Regional Hospital, Tallinn, Estonia

## Abstract

**Background:**

Human linkage and animal QTL studies have indicated the contribution of genes on Chr17 into blood pressure regulation. One candidate gene is *PNMT*, coding for phenylethanolamine-N-methyltransferase, catalyzing the synthesis of epinephrine from norepinephrine.

**Methods:**

Fine-scale variation of *PNMT *was screened by resequencing hypertensive (n = 50) and normotensive (n = 50) individuals from two European populations (Estonians and Czechs). The resulting polymorphism data were analyzed by statistical genetics methods using Genepop 3.4, PHASE 2.1 and DnaSP 4.0 software programs. *In silico *prediction of transcription factor binding sites for intron 1 was performed with MatInspector 2.2 software.

**Results:**

*PNMT *was characterized by minimum variation and excess of rare SNPs in both normo- and hypertensive individuals. None of the SNPs showed significant differences in allelic frequencies among population samples, as well as between screened hypertensives and normotensives. In the joint case-control analysis of the Estonian and the Czech samples, hypertension patients had a significant excess of heterozygotes for two promoter region polymorphisms (SNP-184; SNP-390). The identified variation pattern of *PNMT *reflects the effect of purifying selection consistent with an important role of PNMT-synthesized epinephrine in the regulation of cardiovascular and metabolic functions, and as a CNS neurotransmitter. A striking feature is the lack of intronic variation. *In silico *analysis of *PNMT *intron 1 confirmed the presence of a human-specific putative Glucocorticoid Responsive Element (GRE), inserted by *Alu*-mediated transfer. Further analysis of intron 1 supported the possible existence of a full Glucocorticoid Responsive Unit (GRU) predicted to consist of multiple gene regulatory elements known to cooperate with GRE in driving transcription. The role of these elements in regulating *PNMT *expression patterns and thus determining the dynamics of the synthesis of epinephrine is still to be studied.

**Conclusion:**

We suggest that the differences in PNMT expression between normotensives and hypertensives are not determined by the polymorphisms in this gene, but rather by the interplay of gene expression regulators, which may vary among individuals. Understanding the determinants of PNMT expression may assist in developing PNMT inhibitors as potential novel therapeutics.

## Background

Hypertension is a critical risk factor for cardiovascular disease. Estimates from the studies of familiar aggregation indicate that approximately 30% of blood pressure variance is due to a genetic component [1]. Human Chr17 harbors genes possibly playing an important role in blood pressure regulation [2]. Multiple evidence from the genetic analysis of hypertensive rats indicates that genes on Chr10, syntenic to human Chr17, might be implicated in the aetiology of hypertension [3,4]. Consistently, there is a group of markers at an interval 60–67 cM from the proximal telomere on Chr17, that has been reported to demonstrate significant evidence of linkage in human families with clustering of essential hypertension [2,4]. Furthermore, for four pseudohypoaldosteronism type II (PHAII; Gordon's syndrome) pedigrees characterized by Mendelian inheritance of the disease, four different mutations, possibly leading to increased salt reabsorption and intravascular volume were found in *PRKWNK4 *(WNK lysine deficient protein kinase 4) at 17q21-22 [5].

Angiotensin Converting Enzyme (*ACE*) is the only Chr17 candidate gene investigated in detail for the role in the aetiology of essential hypertension. Still, association studies targeted to *ACE *polymorphisms are inconsistent about the role of *ACE *variants providing susceptibility to hypertension [1]. Another functionally relevant candidate at 17q21-22 is *PNMT*, coding for phenylethanolamine-N-methyltransferase, which has, however, attracted less attention as an affecter of human blood pressure. PNMT catalyzes the synthesis of epinephrine from norepinephrine, the last step of catecholamine biosynthesis. Although, it is mainly expressed in neuroendocrine chromaffin cells in the adrenal medulla, extraadrenal PNMT has been suggested to be involved in the development of hypertension in rat [6]. In human phaeochromocytomas, catecholamine-producing neuroendocrine tumours arising from chromaffin cells or extra-adrenal paraganglian, 50–60% of patients suffer from sustained and 30% from paroxysmal hypertension [7]. Inhibitors of PNMT reduce its activity in the brainstem and have reported to lower blood pressure in the spontaneously hypertensive rats (SHR) [8]. *Pnmt *mRNA expression in Central Nervous System (CNS) was significantly greater in SHR compared to normotensive strains, and was positively correlated with systolic blood pressure [9]. However, comparative sequencing of the rat *Pnmt *coding regions has revealed no sequence differences between stroke-prone spontaneously hypertensive rat (SHRSP) and normotensive Wistar-Kyoto rat genes [10]. Although *Pnmt *expression, its regulation, and involvement in blood pressure maintenance have been intensively studied for animal (rat, bovine) models [11], there is a scarce of knowledge about human *PNMT *gene. The early works on cloning of the human *PNMT *gene [12,13] revealed that two types of mRNA transcripts are produced through the use of alternative promoters. To our knowledge the only published study focusing on *PNMT *gene and hypertension investigated the role of two 5'upstream Single Nucleotide Polymorphisms (SNPs) and reported a significant enrichment of the G-allele of *PNMT*-390 promoter variant for hypertensive African Americans, but not for the Greeks and Americans of European decent [14]. We aimed (1) to resequence the entire human *PNMT *gene in hypertensive and normotensive individuals of European origin in order to uncover the fine-scale sequence variation and to identify novel hypertension-susceptibility polymorphisms; (2) to apply population genetics statistics and *in silico *methods to trace the evolutionary pressure on human *PNMT *and to explore a gene regulatory potential of *PNMT *first intron reflecting the effect of purifying selection.

## Methods

### DNA samples of hypertensive and normotensive individuals

The study has been approved by the Ethics Committee on Human Research of University of Tartu, Estonia (permission no 122/13,22.12.2003). All recruited individuals gave their informed consent prior to their inclusion in the study. Diagnosis and classification of hypertension was carried out by "Practice Guidelines for primary care physicians: 2003 ESH/ESC hypertension Guidelines" Journal of Hypertension 21(10)" pp. 1779–1786. Estonian individuals with primary hypertension (n = 25) were recruited into the study by blood pressure specialists (cardiologist M.V.; nephrologist M.O.) during their ambulatory visit or hospitalization in the Cardiology and Internal Medicine Clinics at the Tartu University Hospital, Estonia. Czech individuals with essential hypertension (n = 25) were recruited from the Cardiology Department of the 2^nd ^Clinic of Internal Medicine, Faculty Hospital Královské Vinohrady in Prague (coordinated by V.K.). Details of the collection of Czech patients and normotensives are published elsewhere [15]. The criteria for the selection of hypertensive individuals for the study:

1. Hypertension diagnosed by a specialist (cardiologist, nephrologist)

2. Grade 2 or severe hypertension at diagnosis (systolic blood pressure, SBP >160 mmHg; diastolic blood pressure, DBP >100 mmHg)

3. Age at diagnosis for men ≤55 and women ≤65 years (to exclude secondary, age-related hypertension)

4. Exclusion of secondary causes for hypertension (such as diabetes, primary renal failure) by a specialist clinician

5. Family background of high blood pressure (to maximize genetic susceptibility)

Hypertension was documented among 1^st ^degree relatives for 84% (n = 21) of Estonian and 92% (n = 23) of Czech patients and among 2^nd ^degree relatives for 28% (n = 7) of Estonian hypertensives (for Czech: data not available). Multiple hypertensive family members were reported for 36% (n = 9) of Estonian and 48% (n = 12) of Czech patients.

6. All recruited individuals received antihypertensive treatment prescribed by a specialist. Blood pressure measurements under antihypertensive therapy were documented: SBP mean 142.8 and range 115 – 215 mmHg; DBP mean 88.4 and range 80–105 mmHg).

A classical case-control study maximizes its power by sampling a clinically homogeneous group of patients. The aim of our resequencing and mutation screening study was to obtain exhaustive coverage of *PNMT *variation in hypertensives. Thus, a high proportion of included patients (Estonians 56%; Czech 92%) exhibited a spectrum of heterogeneous high-blood pressure related organ complications (MI, CAD, stroke, end-stage renal disease).

Normotensives (age at recruitment >35 years) exhibited age-adjusted normal blood pressure based on repeated measurements, lack of family history of essential hypertension, absence of organ damages and had never been prescribed antihypertensive medication. Estonian normotensives (n = 25) were recruited in collaboration with Estonian Blood Centers across Estonia, including only long-term blood donors exhibiting optimal (<120/<80 mmHg)/normal (120–129/80–84 mmHg) blood pressure. Hypertensive and normotensive groups did not differ in Body Mass Index (BMI), a risk factor for hypertension (Mann-Whitney U-test; p > 0.05, two-tailed). BMI ranged for Estonian hypertensives 28.7 ± 6.3, and normotensives 25.2 ± 3.0; and for Czech hypertensives 26 ± 2.3, and normotensives 24.6 ± 2.5.

### Re-sequencing the human PNMT gene

The sequence of human *PNMT *gene [GenBank:J03280.1] has been obtained from NCBI GenBank database [16]. PCR and sequencing primers for *PNMT *(Table 1) were designed using the web-based Primer3 software [17]. The uniqueness of all the primers was checked using BLAST [18]. The PCR primers were designed to cover the entire coding region and parts of 5' and 3' UnTranslated Regions (UTR) (3148 bp); and amplify two overlapping PCR fragments, 1846 bp and 1842 bp respectively. Amplification was performed with 100 ng genomic DNA using Long PCR Enzyme Mix (MBI Fermentas). Conditions for PCR amplifications, product purification, sequencing, sequence contig assembly and polymorphism identification are described in detail elsewhere [19].

**Table 1:** PCR and sequencing primers for human *PNMT *gene

Primers	Sequence 5'-3'
PCR primers for the first and second gene fragments	
PNMT_PCR1_F	AACCCGAACCTTCTGTCCTC
PNMT_PCR1_R	CAGAGTTAGACTGAACCCAGCTC
PNMT_PCR2_F	GCTCAGAATTGAGAGCTAAGGTG
PNMT_PCR2_R	TGTTTGTGACTTCACCTCTCTGA
Sequencing primers for the first PCR fragment	
PNMT_seq1_F	CTAAGTGCATTAGCACAGCTCAC
PNMT_seq1_R	ATCCTCCCCACCCATTCATC
PNMT_seq2_F	GTCTAAAGATTGTGGGGGTGAG
PNMT_seq2_R	CTCTCCTAAGGGATGTTGCTCTT
PNMT_seq3_F	ACGAGGGACAAGAGGTCGT
PNMT_seq3_R	GTGGATCCTAAGGTTGGGAGTT
Sequencing primers for the second PCR fragment	
PNMT_seq4_F	ATAGGAGGAAATGGAGGCAGA
PNMT_seq4_R	CCTGAACCAATGTCGATGAG
PNMT_seq5_F	TTGCAGAGGAGAAGGAAGAACTA
PNMT_seq5_R	TCAGCAGCGTGGTGATGT
PNMT_seq6_F	TGCTGGCAGGATAAGGAG
PNMT_seq6_R	AAAAAGCCTAGGGTGAATGTCTC

### Data analysis

Testing of Hardy-Weinberg equilibrium (α = 0.05); estimation of allele and genotype frequencies of identified Single Nucleotide Polymorphisms (SNPs); and comparison of allelic/genotypic as well as promoter haplotype distribution between hypertensives and normotensives (Fisher's exact test; α = 0.05) were implemented with Genepop software (Version 3.4) [20]. Promoter haplotypes were predicted by the Bayesian statistical method in the program PHASE 2.1 [21].

Sequence diversity parameters were calculated by DnaSP software (Version 4.0) [22]. Nucleotide diversity provides a measure of genetic variation that is normalized by the number of sampled chromosomes. We calculated two conventional measures of nucleotide diversity: (i) π, the direct estimate of per-site heterozygosity derived from the observed average pairwise sequence difference and (ii) Watterson's θ [23], the population mutation parameter representing an estimate of the expected per-site heterozygosity based on the number of segregating site (S). Tajima's D statistic (D^T^) [24] was calculated to determine if the observed pattern of human *PNMT *gene diversity is consistent with the standard neutral model. The D^T ^value is the difference between π and θ estimates. In case of neutrality π equals θ, and thus D^T ^statistic equals zero. The direction of D^T ^statistics (either <0 or >0) is potentially informative about the evolutionary and demographic forces that the population has experienced. Significant positive D^T ^values indicate an excess of intermediate-frequency alleles consistent with either balancing selection or population bottleneck, whereas significant negative D^T ^values indicate an excess of rare SNPs consistent with either recent directional selection or an increase in population size.

*In silico *prediction of transcription factor-binding sites (TFBS) for *PNMT *intron 1 was carried out by MatInspector 2.2 [25,26] online software. The program identifies TFBS in nucleotide sequences using large library of position weight matrices (PWM) and is based on the information about experimentally defined TFBSs collected in the TRANSFAC database [27]. The presence of repetitive sequences was analyzed by RepeatMasker online software [28].

## Results

### Resequencing of human PNMT reveals low diversity

**Figure 1:**
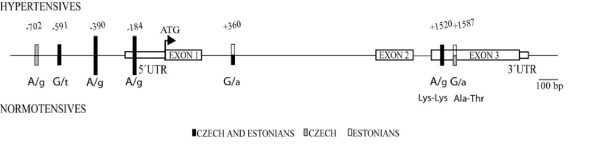
The structure of human *PNMT *gene drawn to an approximate scale. The identified SNPs (major allele by capital letters) are located relative to translation start site (ATG), where A denotes +1. SNPs with minor allele frequency <10% are indicated with short bars and >10% with long bars. Variants detected both in Estonians and Czech are shown in black; only in Czech are in grey and only in Estonians in white. The SNPs above and below the graph represent polymorphisms found in hypertensive and normotensive individuals, respectively. The 5'and 3'UTR have been indicated according to NCBI GenBank database (February 28, 2006 release).

We resequenced the human *PNMT *genomic sequence (in total 3187 bp) in hypertensive (n = 50) and normotensive (n = 50) individuals originating from two European populations. The analyzed region (from -882 to +2305 relative to ATG) included the entire 5' and 3'UTR regions, three exons (424 bp, 208 bp and 525 bp respectively) and two introns. The identified diversity patterns supported the conservative nature of the *PNMT *gene. We determined only three rare genic polymorphisms; in contrast, the upstream region harboured four SNPs, two of which (SNP -184 A/G; SNP -390 A/G) are previously characterized common variants (Table 2, Figure 1). Majority of the SNPs were identified in both populations and none was exclusively present in only hypertensive individuals. Compared to a data set of 74 genes [29], characterized by a mean nucleotide diversity parameter π = 0.00080 for European Americans, *PNMT *gene exhibited approximately three times lower diversity among studied populations including both normotensive and hypertensive individuals (π = 0.00026 – 0.00032; Table 3). As the only identified non-synonymous mutation (Exon 3; SNP +1517; Ala->Thr) was present in both populations for individuals with normal (Major Allele Frequency, MAF = 6%) as well as elevated (MAF = 4%) blood pressure, we exclude this protein variant increasing susceptibility for hypertension.

**Table 2:** Allele (major) and genotype (major homozygote, heterozygote) frequencies for identified SNPs by resequencing in Estonian and Czech normotensives and hypertensives

SNP^a^	dbSNP	Allele^b^/Genotype	Estonians		Czech	
			Normotensives	Hypertensives	Normotensives	Hypertensives
SNP-702	NA	G	1	1	0.96	0.98
5'UTR		GG	1	1	0.92	0.96
		GA	0	0	0.08	0.04
SNP-591	NA	G	0.96	0.96	0.98	0.88
5'UTR		GG	0.92	0.92	0.96	0.88
		GT	0.08	0.08	0.04	0
SNP-390	NA	A	0.60	0.70	0.85	0.675
5'UTR		AA	0.44	0.40	0.79	0.45
		AG	0.32	0.60	0.125	0.45
SNP-184	rs876493	A	0.60	0.58	0.66	0.54
5'UTR		AA	0.40	0.24	0.44	0.20
		GA	0.40	0.68	0.44	0.68
SNP+360	rs200173	G	0.98	1	0.96	0.96
Intron 1		GG	0.96	1	0.92	0.92
		AG	0.04	0	0.08	0.08
SNP+1520	rs5638	A	0.94	0.96	0.94	0.96
Exon 3		AA	0.92	0.92	0.88	0.92
		AG	0.04	0.08	0.12	0.08
SNP+1587	NA	G	0.98	1	1	0.98
Exon 3		GG	0.96	1	1	0.96
		AG	0.04	0	0	0.04

^a^location relative to ATG; ^b^major allele frequency; NA-not available

When we addressed the population differentiation of the identified SNPs by Fisher's exact test, none of the polymorphisms showed either allelic or genotypic differentiation among the Estonians and the Czech, as well as between normotensive and hypertensive individuals in the intrapopulation comparisons (p > 0.05, data not shown). The joint case-control analysis of all samples with an increased test power detected a significant excess of heterozygotes for common promoter region polymorphisms (SNP-184; SNP-390) among the patients (Fisher exact test, p < 0.05; Table 2). There was a non-significant difference between normotensives and hypertensives for the distribution of haplotypes formed from two common 5'UTR polymorphisms, SNP -390 and SNP -184.

However, the most striking feature of PNMT gene is the lack of variation within introns harboring only one rare SNP (Figure 1; intron 1, SNP+360). The introns exhibit >15-fold less diversity compared to analyzed 5'upstream region (π_intron _= 0.00002 – 0.00007; π_5'upstream _= 0.00099–0.00138; Table 3) and >7-fold reduction in diversity compared exons (π_exon _= 0.00050–0.00056). In comparison, the excess of diversity for the 5'upstream region compared to the exons is only 2–2.5 times. To test whether patterns of DNA sequence variation in PNMT fit the expectations under hypothesis of neutrality, we analyzed the data with the Tajima DT neutrality test  (Table 3). When the analysis included an entire PNMT gene there was no difference between observed (π) and expected (θ) nucleotide diversity parameters. Notably, intronic sequences formed an exception with the expected variation (θ = 0.00018–0.00021) exceeding the observed values (π = 0.00002–0.00007) up to tenfold. Whereas the lack of diversity in intron 2 spanning only 114 bp is apparently due to functional constraint on neighboring exons, minimal variation and consistent Tajima DT values for intron 1 support an effect of purifying selection on this region.

**Table 3:** Sequence diversity parameters of human *PNMT *gene region

			Estonians^d^			Czech^d^		
	kb		Hyper	Normo	All	Hyper	Normo	All
Sequenced area	3.187	π^a^	0.00035	0.00039	0.00037	0.0030	0.00024	0.00027
		θ^b^	0.00035	0.00042	0.00036	0.00042	0.00035	0.00036
		D^c^	0.01342	-0.16433	0.04368	-0.72287	-0.75219	-0.56268
Gene	2.222	π	0.00026	0.00031	0.00028	0.00032	0.00029	0.00031
		θ	0.00020	0.00040	0.00035	0.00040	0.00030	0.00035
		D	0.51545	-0.52876	-0.37972	-0.48106	-0.05656	-0.24179
Exons	1.157	π	0.00050	0.00056	0.00052	0.00054	0.00050	0.00052
		θ	0.00039	0.00058	0.00050	0.00058	0.00039	0.00050
		D	0.51545	-0.07671	0.07805	-0.13715	0.50566	0.06616
Introns	1.065	π	NA	0.00004	0.00002	0.00007	0.00007	0.00007
		θ	NA	0.00021	0.00018	0.00021	0.00021	0.00018
		D	NA	**-1.10280**	**-1.02786**	**-0.87191**	**-0.87191**	**-0.68607**
5'upstream region	0.882	π	0.00114	0.00120	0.00116	0.00138	0.00099	0.00121
		θ	0.00076	0.00076	0.00066	0.00101	0.00101	0.00088
		D	1.03138	1.19760	1.38242	0.83180	-0.03929	0.74751

Estimate of nucleotide diversity per site from ^a^average pairwise difference among individuals and ^b^number of segregating sites (S); ^c^Tajima's D statistics; ^d^n = 25 for normotensives, n = 25 for hypertensives; NA – not applicable

Alignment of human *PNMT *and rat *Pnmt *gene reveals high level of exonic conservation (>82%) at the level of DNA sequence between two species (See additional file 1). In contrast to the expectations based on selection pressure shown for human intron 1, there is no homology between intron 1 of *PNMT *and *Pnmt *genes. An *AluSp *insertion in primate lineage (See additional file 1) has changed the genomic landscape of human *PNMT*.

### In silico analysis of PNMT intron 1 for putative binding sites of gene regulatory elements

**Figure 2:**
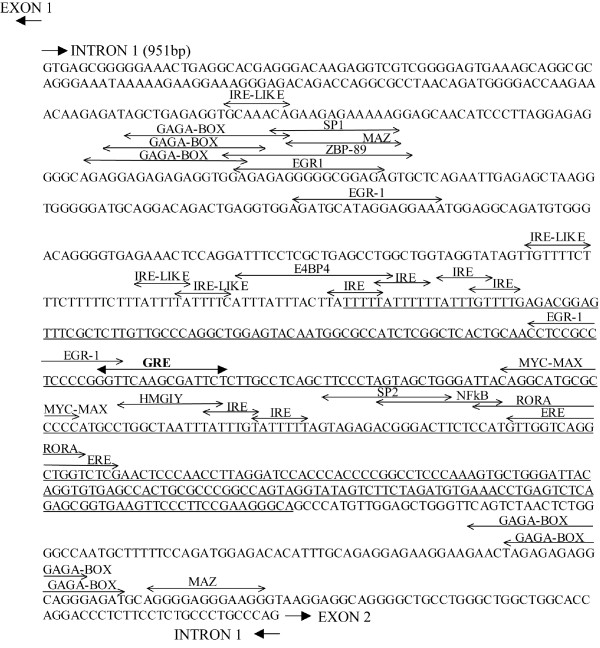
Identification of putative transciption regulating elements within human *PNMT *intron 1. Putative transcription factors binding sites (TFBS) predicted by MatInspector 2.2 software and regulatory elements identified by manual inspection are depicted upon the sequence of *PNMT *intron 1 (951 bp). The Glucocorticoid Responsive Element (GRE; consensus GGTACAnnnTGTTCT), a core for a potential Glucocorticoid Responsive Unit (GRU), is given in bold. The human-specific *AluSp *element is underlined. The two-directional arrows indicate the predicted binding sites for regulatory factors: IRE – Insulin Responsive Element (consensus T(G/A)TTT(T/G)(G/T)); ERE – Estrogen Responsive Element (consensus GGTCAnnnTGACC); NFκB – Nuclear Factor kappa B; Sp1/2 – Specificity protein 1/2; Egr1 – Early Growth Response 1; MAZ – Myc-Associated Zinc finger protein; ZBP-89 – Zinc finger Binding Protein 148, ZNF148; HMGI/Y – High Mobility Group protein isoform I and Y, HMGA1; RORA (RORα) – Retinoic acid receptor-related Orphan Receptor α; E4BP4 – mammalian transcription factor E4 Binding Protein 4.

In order to explore the hypothesis of the purifying selection acting on human *PNMT *intron 1, we analyzed *in silico *the distribution of functionally important putative regulatory elements in this region. Two seminal works [12,13] have predicted the presence of a Glucocorticoid Responsive Element (GRE) in the middle of intron 1. In a number of genes, the glucocorticoid (GC) response is enhanced by the binding of other transcription factors to adjacent binding sites, forming Glucocorticoid Responsive Units (GRUs) [30]. We explored putative *PNMT *regulatory elements among the predicted transcription factor binding sites (TFBS) within intron 1 (Figure 2). We focused on TFBS-s either reported (1) to regulate rat *Pnmt *gene; (2) to form active GRUs; (3) to locate within intronic gene regulatory units; or (4) selected as potential inducers/repressors of epinephrine synthesis.

In rat, *Pnmt *transcription is synergistically activated by binding of Egr-1 (Early Growth Response 1), AP2 (Activating enhancer binding Protein 2 alpha) and GR (Glucocorticoid-activated Receptor complex) to the upstream promoter [31], whereas Sp1 (Specificity protein 1) and MAZ (Myc-Associated Zinc finger protein) transcription factors may potentially contribute to tissue-specific expression [32]. In human *PNMT *intron 1 we identified multiple binding sites for Egr1, MAZ and Sp1/Sp2 (Figure 2). A shared recognition site for Egr1/MAZ/Sp1 276 bp upstream GRE overlaps with a binding site for another zinc-finger nuclear protein – ZBP-89 (Zinc finger Binding Protein 148). For example, for bovine adrenodoxin gene expressed in adrenal cortex, Sp1/Sp3 confer and ZBP-89 represses basal transcriptional activities [33]. An alternative binding site for Sp1/Sp2 (74 bp downstream GRE) is co-localized with binding site for NFκB (Nuclear Factor kappa B), a transcription factor involved mainly in inflammatory and immune responses. There is an overlap also in the function of the two elements since GCs have potent anti-inflammatory and immunosuppressive properties. NFκB activity is antagonized by GCs either directly through inhibiting NFκB binding to DNA or indirectly by binding of the hormone-activated GC receptor complex (GR) to GRE [34].

The most striking feature of human *PNMT *intron 1 is the abundance of predicted Insulin Responsive Elements [IRE, consensus sequence T(G/A)TTT(T/G)(G/T)]. GRE element is flanked both sides by multiple overlapping perfect IREs (four and two, respectively). Additional four IREs differing by one nucleotide from the consensus [T(G/A)TTT(T/G)C] are located in the vicinity. It has been shown that for a subset of genes insulin inhibits GR activity either via IRE-dependent or IRE-independent mechanisms [35]. *PNMT *intron 1 distal IREs overlap with a predicted binding site for HMGI/Y (High Mobility Group protein isoform I and Y), a mammalian architectural transcription factor that participates in specific protein-DNA and protein-protein interactions that induce both structural changes in chromatin substrates and the formation of stereospecific multiprotein complexes on the promoters/enhancers of genes whose transcription they regulate [36]. This factor is also part of nucleoprotein complex controlling human insulin receptor gene transcription and its loss causes insulin resistance and diabetes in human and mice [37].

Among the other reported GRU-belonging *cis*-acting elements, we identified (i) binding sites for Myc/Max family proteins, reported for the functional GRU of ovine β1-adrenergic receptor gene [38]; (ii) two regions of overlapping putative GAGA-boxes shown to increase the rate of GRE-induced gene expression in the proximal enhancer of human carbamoylphosphate synthetase gene [39]; (iii) and Estrogen Responsive Element (ERE) [40]. Interestingly, ERE within the first intron of *PNMT *co-localizes with a binding site for RORA (Retinoic acid receptor-related Orphan Receptor α; also called RORα), reported among the key regulators of mammalian circadian gene expression [41]. Another putative 'clock controlled element', the binding site for a light-induced transcriptional repressor *E4BP4 *(mammalian transcription factor E4 Binding Protein 4) [42], was predicted within the cluster of IREs proximal to GRE.

## Discussion

Resequencing revealed low variation of *PNMT *gene in both normotensive and hypertensive individuals consistent with an important role of PNMT-synthesized epinephrine in the regulation of cardiovascular and metabolic function and as a CNS neurotransmitter. Our data on human *PNMT *agrees with the report on comparative sequencing of the rat *Pnmt *in hypertensive SHRSP and normotensive strains [10] and allows to suggest that most of high between-subject variation in PNMT-synthesized epinephrine levels [43] does not result from *PNMT *gene variants. Indeed, the only described human PNMT allozyme displaying significantly lower levels of activity compared to wild-type, is a rare African-American variant Thr98Ala [44]. The expressional variation of *PNMT *gene is rather determined by the interplay of hormonal and neural stimuli as well as by the repertoire of regulating transcription factors [11,45].

Interestingly, the sequence parameters of the genomic region coding for the structurally and evolutionary most related enzyme to PNMT, the glycine-N-methyltransferase (GNMT) resemble the *PNMT *region [46]. Both genes have a similar size (the *GNMT *gene: 3.118 kb) and an exon-intron structure with a long first intron harbouring a SINE element; a low number of common SNPs, reduced diversity (NCBI dbSNP and International HAPMAP Project databases) and strong linkage disequilibrium. This supports the suggested evolutionary relationship between the two genes [46] as well as underlines the conserved role of N-methyltransferases in regulating essential metabolic functions.

Although rat *Pnmt *promoter has been characterized in detail, the regulatory regions of human *PNMT *gene have not been studied, with the exception of two common promoter SNPs. Consistent with a previous report [14], there was no significant difference in allele frequencies for SNP-184 and SNP-390 between European hyper- and normotensives. However, the heterozygote status of these promoter region polymorphisms was significantly associated with the diagnosis of hypertension. Interestingly, for neurological diseases such as Alzheimer disease and multiplex sclerosis, a protective effect of the heterozygous status of both SNPs (-390/-184 GA/AG) has been shown [47,48]. A recent functional study [44] using dual-luciferase assay has shown approximately 30% decrease in reporter gene activity for a construct carrying -390A/-184A haplotype (reported 50–60% frequency in Europeans) compared to -390G/-184G variant (60–70% in Africans).

Intron 1 of human *PNMT *exhibits minimum diversity consistent with purifying selection. Two seminal works [12,13] predicted a putative GRE into this region, but due to a location within an *Alu*-sequence its functionality was questioned at that time [13]. Yet, there are several examples in which *Alu *sequences were inserted into human gene regions long time ago, were modified, and now are central in control/enhancement of transcription [49]. Glucocorticoid (GC) sensitivity has been reported for *PNMT *promoter activity in rat [31] and bovine [50], and at least one putative GRE has been identified for every species-specific *PNMT *gene. We explored a scenario that the *Alu*-introduced human-specific GRE in *PNMT *intron 1 has evolved to a functional GRU with an essential role in gene expression regulation and therefore is under selective constraint. Interestingly, the *PNMT *intronic GRE was surrounded by multiple potential IREs. There are multiple examples of genes (*IGFBP-1, TAT, AAT, PEPCK, PFK-2*) stimulated by glucocorticoids and inhibited by insulin in liver [35]. To our knowledge, insulin responsiveness has not been studied for rat or bovine *Pnmt *promoters. But already years ago it was shown that PNMT activity was approximately 2-fold higher in the brainstem of streptozotocin-induced diabetic rats than in controls, and the administration of insulin partially prevented the effects of diabetes on PNMT activity [51]. In the adrenal medulla of rats, *PNMT *mRNA levels were increased as a response to a single administration of insulin 5I U [52]. A recent study using diabetic rats confirmed a protective effect of insulin treatment on PNMT levels and counter regulation [53] of epinephrine.

The location of gene regulatory elements within intronic regions is not unique. For example, the first intron for rat liver *6-phosphofructo-2-kinase *(*Pfk-2*) gene harbours GRU targeted by both glucocorticoids and insulin [35] and the transcription of mouse c-HA-*ras *gene is jointly regulated by a GRE and ERE (Estrogen Responsive Element) [54] similarly to the prediction for *PNMT *intron 1 GRU. It has been reported that ERβ-deficient mice develop sustained systolic and diastolic hypertension by 5 months of age and males have higher blood pressure than females [55]. Consistently, Peng [56] showed that estrogen depletion increases blood pressure and hypothalamic norepinephrine in middle-aged spontaneously hypertensive rats.

It has been shown that the levels of epinephrine/norepinephrine (greatly regulated by PNMT enzyme) and also cortisol (the most abundant GC) exhibit circadian fluctuation [43]. Consistently, *PNMT *intron 1 GRU harbors potential binding sites for transcription factors regulating mammalian circadian clocks, RORα and E4BP4 [41]. Patients with essential hypertension have disturbed autonomic cardiovascular regulation and circadian pacemaker function [57]. It has been suggested that a changed suprachiasmatic nucleus (SCN), the mammalian central 'clock' within the hypothalamus in the brain, may precede the development of hypertension[58].

## Conclusion

We report low diversity of human *PNMT *gene consistent with purifying selection. We suggest that the differences in *PNMT *expression between normotensives and hypertensives are not determined by the polymorphisms in this gene, but rather by the interplay of gene expression regulators, which may vary among individuals. A major human-specific gene regulatory unit, Glucocorticoid Responsive Unit (GRU), was predicted within *PNMT *intron 1. *In silico *analysis paves the way for the further experimental studies on human *PNMT *transcription regulation. Understanding the determinants of *PNMT *expression may assist in developing PNMT inhibitors as potential novel therapeutics, facilitated by recent determination of the crystal structure of human PNMT[46].

## Competing interests

The author(s) declare that they have no competing interests.

## Authors' contributions

KK designed the study, performed resequencing, and contributed to the analysis and interpretation of the data as well as to the writing of the manuscript. MO, MV and VK recruited essential hypertension patients, collected relevant clinical and epidemiological data, and revised the manuscript since its early versions. PJ assisted in collection of normotensive individuals, preparation of the DNA samples and laboratory experiments. ML contributed in outlining the study design and directing research, participated in the data analysis and drafted the manuscript. All authors read and approved the final manuscript.

## Pre-publication history

The pre-publication history for this paper can be accessed here:



## Supplementary Material

Additional file 1 Nucleotide sequence alignment of human *PNMT *and rat *Pnmt *genes. Exons of the gene are boxed. mRNA start site is marked with +1. Translation start site is indicated by ATG (red/bold) and red arrow; translation end sites for human and rat are in purple/bold. Seven SNPs identified by resequencing *PNMT *in this study are marked bold with grey shading. The sequence of human-specific *AluSp element *in the first intron is marked in italics and its start/end sites are indicated by black arrows. Sequences of regulatory elements and transcription factor binding sites (TFBS) are underlined. Experimentally confirmed rat *Pnmt *expression regulating elements [6,59-62] are typed in *italics *on green shading background. Predicted regulatory elements for human *PNMT *[12,13] are typed with regular font (no shading). Click here for file
