# Supplementary material for: Resequencing PNMT in European hypertensive and normotensive individuals: no common susceptibilily variants for hypertension and purifying selection on intron 1
Source: BMC Med Genet. 2007 Jul 23;8:47. doi: 10.1186/1471-2350-8-47 (PMC1947951; doi:10.1186/1471-2350-8-47)
Supplement: Additional file 1 — Nucleotide sequence alignment of human PNMT and rat Pnmt genes. Exons of the gene are boxed. mRNA start site is marked with +1. Translation start site is indicated by ATG (red/bold) and red arrow; translation end sites for human and rat are in purple/bold. Seven SNPs identified by resequencing PNMT in this study are marked bold with grey shading. The sequence of human-specific AluSp element in the first intron is marked in italics and its start/end sites are indicated by black arrows. Sequences of regulatory elements and transcription factor binding sites (TFBS) are underlined. Experimentally confirmed rat Pnmt expression regulating elements [6,59-62] are typed in italics on green shading background. Predicted regulatory elements for human PNMT [12,13] are typed with regular font (no shading). [file 1471-2350-8-47-S1.pdf]

Additional files

Additional file 1

File format: .pdf

Title and description of the data:

Nucleotide sequence alignment of human *PNMT* and rat *Pnmt* genes. Exons of the gene are boxed. mRNA start site is marked with +1. Translation start site is indicated by **ATG** (red/bold) and **red** arrow; **translation end sites** for human and rat are in purple/bold. Seven **SNPs** identified by resequencing *PNMT* in this study are marked bold with grey shading. The sequence of human-specific *AluSp element* in the first intron is marked in italics and its start/end sites are indicated by **black** arrows. Sequences of regulatory elements and transcription factor binding sites (TFBS) are underlined. Experimentally confirmed rat *Pnmt* expression regulating elements [6, 59-62] are typed in *italics* on green shading background. Predicted regulatory elements for human *PNMT* [12-13] are typed with regular font (no shading).

```
hPNMT    1 TTCAGGAAGACCACGGAGATGCCACAGGCAGAATTGGCTTCCCATCTGGGAGATAGGTGG
rPnmt    1 --------------------------CT.CAGCCC..T..G..--..A.T.C..G..C..

hPNMT   61 GGAGACCCTGGCATTTTGACAGCCAGAACCTGGGGTGCTGAGCAGAATCTTCATGCCTGG
rPnmt   33 .A....AAGCA....A..-TGC.AG.C....A.A.CA.CA...G.G.C..GA.G...A..

hPNMT  121 CCTGGCCGCCTTCGGAGGGAAGCTGGAGGGTTGGGTGCGAGAGGAGTGGGGTCAGAGCCC
rPnmt   92 ..CT...AA..C.CC..T.GGA.A...T.-...AC.--...G..T.G.....G.AG.ATG

hPNMT  181 CTACATCCGCAGGACCCCAAATCGGCTGGGCCCCAAGGCCCGGAC-TGCGCT-CCCCGGT
rPnmt  149 --------.....GT..TCT.C.T.TGAAAAA....AAAGT.CG.A......G..T.T..
                                                GRE
hPNMT  239 GGCCCCGGCGGCCCT-CCGCGAATGCGTCCTGCCCCTCCCCTGCCCAAGCCCTCTGCCCT
rPnmt  201 A.TGG.T.T...AG.A..AA.....T..T....----------------A.T.....TT..
                                                                GRE
hPNMT  298 CACCCGGGTCCGGCGCCGCCCCCGAAGTGGCGGGAACAACCCGAACCCGAACCTTCTGTC
rPnmt  246 T..A..A......T.T---...T..CC...TA......CTT.....T--....----A.G
                                                      AP2
hPNMT  358 CTCGGGAGCCCCCAGATAAGCGGCTGGGAACCCGCGGGGCCCGCAGGGGAGGCCCGGCTG
rPnmt  297 ..T.CCG.A..T.........A..GCAT.G...-.A......A.....-.T......A..
                              NicRe                          NicRe
hPNMT  418 TTCCGCCCGCTAAGTGCATTAG-CACAGCTCACCTCCCCTATCGCGCCTGCCATCGGACG
rPnmt  355 ...T..TT.....AA.......A.C.CA.......G---....T.TG........AA.T.
                                                                  GRE
hPNMT  477 GGCAGTGCCGCGCCCTGCTCTGGGGCCCCCGGAGCGACCACAGCGGAGGCCGGAACGGAC
rPnmt  412 CT.G.G..A.A.A.......---A.TA...A.-..C....AGA.A......A....A..G
                                     Sp1
hPNMT  537 TGTCCTTTCTGGGGCGGGGTGGGGAGGGGGTGTCGCTGGAGGGCCCGGTGGCATAGCAAC
rPnmt  468 ...........AA.GA..A.A.A..C.....------------------------------

hPNMT  597 GGACGAGAGAGGCCTGGAGGAGGGGCGGGGAGGGGAGTTGTGTGGCAGTTCTAAGGGAA
rPnmt  499 -------.....T.......T....-----------------A.....-.GA...A.A.G

hPNMT  657 GGGTGGGTGCTGGGACGGGTGTCCGGGAGGGAGGGGAGCCTGGCGGGGTCTGGGGCCTCG
rPnmt  534 ...A.TT..T---A.A....AC..C.AGA..G.A....---------------------.

hPNMT  717 TCGCGGAGGGCGCTGCGAGGGGGAAACTGGGGAAAGGGCCTAATTCCCCAGTCTCCACCT
rPnmt  570 C..G.A...AT....G..TC.....CA..A.-----------------.T..CTCAG....

hPNMT  777 CGAATCAGGAAAGAGAAGGGGCGGGCTGCTGGGCAAAAGAGGTGAATGGCTGCGGGGGGC
rPnmt  614 ----.TG.............AT...A......A.GCTG..C.-..G.--...T...A...

hPNMT  837 TGGAGAAGAGAGATGGGAGGGGCCGGCCGGCGGGGGTGAGGGGGTCTAAAGATTGTGGGG
rPnmt  667 ......G...TC.-...G.CCA.T..--AA.T.A..CTG....AG.AG.GC.GCACT..A
                                               Sp1          Sp1
hPNMT  897 GTGAGGAACTGAGGGTGGGGGGCGCCCAGAGGCGGGACTCGGGGCGGGGCAGGCGAGGCG
rPnmt  724 .C..TC..------------.A..C.AT.A...T..A-----------...TT.T.A..--C
                                                           +1  human exon 1
hPNMT  957 GAGGGCGAGGGCTGCGGGAGCAAGTACGGAGCCGGGGGTGTGGGGGACGATTGCCGCTGC
rPnmt  761 A..A.TA.A.----.A....TGG.-G.A.GAG..AT.T.C.AAA..G..CCCT..A.AT.
                      Egr-1         Sp1
hPNMT 1017 AGCCGCCGCCCCACTCACCTCCGGTGTGTCTGCAGCCCGGACACTAAGGGAGATGGATGA
rPnmt  816 TC..C..T....G.C.-..G.GC..CC...C.GC..T.A........CT..........-

hPNMT 1077 ATGGGTGGGGAGGATGCGGCGCACATGGCCCCGGGCGGCTCGGCGGTCAGCTGCCGCCCC
rPnmt  874 --.....ACAGA....T....GC.TC...G..TCATCC...A..A.C..C.CA..C.TGT
                     Sp1          MAZ
hPNMT 1137 CACAGCGGA--CCGGTCGGGGCGGGGG---TCGGGCGGTAGAAAAAAGGGCCGCGAGGCG
rPnmt  932 G.TG.A..GGT.T..G.....G..A..GGAC.CA.TA.....T...G..ATG.G....TT
```

```
hPNMT 1192 AGCGGGGCACTGGGCGGACCGCGGCGGCAGCATGAGC---GGCGCAGACCGTAGCCCCAA
rPnmt  992 .....A.--A.A.....-..T.AA.A.G......GA.CGT...T......CG.AG.A..C
           +1 rat exon 1

hPNMT 1249 TGCGGGCGCAGCCCCTGACTCGGCCCCGGGCCAGGCGGCGGTGGCTTCGGCCTACCAGCG
rPnmt 1049 ...A..GATG.A.T.......C.A...T........A.A...C..C.T...T........

hPNMT 1309 CTTCGAGCCGCGCGCCTACCTCCGCAACAACTACGCGCCCCCTCGCGGGGACCTGTGCAA
rPnmt 1109 ...T.....C..T..........................T.....T.........A....

hPNMT 1369 CCCGAACGGCGTCGGGCCGTGGAAGCTGCGCTGCTTGGCGCAGACCTTCGCCACCGGTGA
rPnmt 1169 ...TG.T..T..G.....T........T......A....A..AGT...T...........

hPNMT 1429 GCGGGGGAAACTGAGGCACGAGGGACAAGAGGTCGTCGGGGAGTGAAAGCAGGCGCAGGG
rPnmt 1229 ..ACT......A..........A..GC...A...A.A...A.A.....-------.G...

hPNMT 1489 AAATAAAAAGAAGGAAAGGGAGACAGACCAGGCGCCTAACAGATGGGGACCAAGAAACAA
rPnmt 1282 .G.G.G...C...A-----------------.T.TGC.C..A.......A.C.A...GC.

hPNMT 1549 GAGATAGCTGAGAGGTGCAAACAGAAGAGAAAAAGGAGCAACATCCCTTAGGAGAGGGGC
rPnmt 1325 ..AC..T---..GT...T.GC.G.C.C.A.C.----------------------------

hPNMT 1609 AGAGGAGAGAGAGGTGGAGAGAGGGGGCGGAGAGTGCTCAGAATTGAGAGCTAAGGTGGG
rPnmt 1354 -----------------------..A.....CGA.AG..----...GCTA...G..AT..

hPNMT 1669 GGATGCAGGACAGACTGAGGTGGAGATGCATAGGAGGAAATGGAGGCAGATGTGGGACAG
rPnmt 1387 ...G..------------------------.T......C.----A.T..T..A..CC.G.

hPNMT 1729 GGGTGAGAAACTCCAGGATTTCCTCGCTGAGCCTGGCTGGTAGGTATAGTTGTTTTCTTT
rPnmt 1419 AAT..GA.G...----------------------.A..A.ACAACT..A--.G..CT...
                                                  Alu sequence
hPNMT 1789 CTTTTTCTTTATTTTATTTTCATTTATTTACTTATTTTATTTTTTATTTGTTTTGAGAC
rPnmt 1455 ....--------------------------------------------------------

hPNMT 1849 GGAGTTTCGCTCTTGTTGCCCAGGCTGGAGTACAATGGCGCCATCTCGGCTCACTGCAAC
rPnmt 1459 -------------------.....--....G.----------------------------

                                   GRE
hPNMT 1909 CTCCGCCTCCCCGGGTTCAAGCGATTCTCTTGCCTCAGCTTCCCTAGTAGCTGGGATTAC
rPnmt 1470 ------------------------------...T.A.AG..GAGAGAC..A.....CC-T

hPNMT 1969 AGGCATGCGCCCCCATGCCTGGCTAATTTATTTGTATTTTTAGTAGAGACGGGACTTCTC
rPnmt 1499 G..AGA....----------..-------------------GAGG.G..T....------

hPNMT 2029 CATGTTGGTCAGGCTGGTCTCGAACTCCCAACCTTAGGATCCACCCACCCCGGCCTCCCA
rPnmt 1524 ---.....--..A...------------------------------------------..

hPNMT 2089 AAGTGCTGGGATTACAGGTGTGAGCCACTGCGCCCGGCCAGTAGGTATAGTCTTCTAGAT
rPnmt 1537 ...A.A-.A.GC.C..------------------------------------.C..T...
                                        Alu sequence
hPNMT 2149 GTGAAACCTGAGTCTCAGAGCGGTGAAGTTCCCTTCCGAAGGGCAGCCCATGTTGGAGCT
rPnmt 1560 ..C..G---.GA....--------------...CA.TA..TT.T.A.------.A.....

hPNMT 2209 GGGTTCAGTCTAACTCTGGGGCCAATGCTTTTTCCAGATGGAGACACATTTGCAGAGGAG
rPnmt 1597 A.T...T.---.GTG....AA..CGA.T------....-....C.CTG.CA...C-----

hPNMT 2269 AAGGAAGAACTAGAGAGAGGCAGGGAGATGCAGGGGAGGGAAGGGTAAGGAGGCAGGGGC
rPnmt 1642 ----...TG..G.G...-------..C.C.AG..A.....GT..C.---........AA.
                                                          exon 2
hPNMT 2329 TGCCTGGGCTGGCTGGCACCAGGACCCTCTTCCTCTGCCCTGCCCAGGTGAAGTGTCCGG
rPnmt 1688 CC....ATA.----------------.C.C........T..A..-.......G.....T..
```

hPNMT 2389 ACGCACCCTCATCGACATTGGTTCAGGCCCCACCGTGTACCAGCTGCTCAGTGCCTGCAG
rPnmt 1731 ..AGGT......T.....C..C............A.A..............C.....TGC

hPNMT 2449 CCACTTTGAGGACATCACCATGACAGATTTCCTGGAGGTCAACCGCCAGGAGCTGGGGCG
rPnmt 1791 ......C....................C...T.............G...........A.T

hPNMT 2509 CTGGCTGCAGGAGGAGCCGGGGGCCTTCAACTGGAGCATGTACAGCCAACATGCCTGCCT
rPnmt 1851 ........GA.....A..A..A......G.......TG....T.....G....T......

hPNMT 2569 CATTGAGGGCAAGGGGTAAGGACTGGGGGGTGAGGGTTGGGGAGGAGGCTTCCCATAGAG
rPnmt 1911 ...C....A........G..A......C--..G.A.C.TCAC.C.GA.TG.------.G.

hPNMT 2629 TGGCTGGTTG-GGGCAACAGAGGCCTGAGCGT--AGAACAGCCTTGAGCCCTGCCTTGTG
rPnmt 1963 C..G..C.G.A....GGAG....A......C.CTG.GGT..T..-......C........

exon 3

hPNMT 2686 CCTCCTGCACAGGGAATGCTGGCAGGATAAGGAGCGCCAGCTGCGAGCCAGGGTGAA**A**CG
rPnmt 2022 ..C....T....A..G.C.........G..A..A........C.....G........G..

hPNMT 2746 GGTCCTGCCCATCGACGTGCACCAGCCCCAGCCCCTGGGTGCTGGGAGCCCAGCTCCCCT
rPnmt 2082 A...T.......T..T......A................A...TC.G...TG..A.....

hPNMT 2806 GCCTGCTGACGC**C**CTGGTCTCTGCCTTCTGCTTGGAGGCTGTGAGCCCAGATCTTGCCAG
rPnmt 2142 .............T.................C................G.....CC.A..

hPNMT 2866 CTTTCAGCGGGCCCTGGACCACATCACCACGCTGCTGAGGCCTGGGGGGCACCTCCTCCT
rPnmt 2202 ...C.G..A...TT..T.T..T.....G..............C.....TA.T......T.

hPNMT 2926 CATCGGGGCCCTGGAGGAGTCGTGGTACCTGGCTGGGGAGGCCAGGCTGACGGTGGTGCC
rPnmt 2262 ..............................T.................AT.T.....T..

hPNMT 2986 AGTGTCTGAGGAGGAGGTGAGGGAGGCCCTGGTGCGTAGTGGCTACAAGGTCCGGGACCT
rPnmt 2322 ......A..........................CT.......T..TG.......A.....

hPNMT 3046 CCGCACCTATATCATGCCTGCCCACCTTCAGACAGGCGTAGATGATGTCAAGGGCGTCTT
rPnmt 2382 T........C.................C.GC..G..T..G.....C........TA....

hPNMT 3106 CTTCGCCTGGGCTCAGAAGGTTGGGCTG**TGA**GGGCTGTACCTGGTGCCCTGTGGCCCCCA
rPnmt 2442 ...T........C......A.A.A.G..CAG.T.-------..**A**G..T.CA.-----TTG

hPNMT 3166 CCCACCTGGATTCCCTGTTCTTTGAAGTGGCACCTAATAAAGAAATAATACCCTGCCGCT
rPnmt 2490 ..TC...A-.C...T.A.CACCC...................T..C.G.C...A..TAT-

hPNMT 3226 GCGGTCAGTGCTGTGTGTGGCTCTCCTGGGAAGCAGCAAGGGCCCAGAGATCTGAGTGTC
rPnmt 2548 ---...T.......T....A.......A.AC.CA..G.TA..AAA..GTC..C...-.CT

hPNMT 3286 CGGGTAGGGGAGACATTCACCCTAGGCTTTTTTTCCAGAAGCTTCCTTGAGGCTAGCATT
rPnmt 2604 TA.AAG.AACCA.TCC.AG.TT.TTCT....GCC.T......C.T.G...A.GCT..T..

hPNMT 3346 CTGTACCACTCATTCTTCCCAAACTAAGGAAGGCCAAGGTCAGGGGAGCTCACCGGGCAC
rPnmt 2664 ....G....C......C...TCC.C.G.-------....C..CAC.CA.GTG..A...G.

hPNMT 3406 CTGCAGTGCACCAGGCACTTGTGATATGCCATTGTGTC
rPnmt 2717 T..TGTG.TT.TC.A.CTGCAG.C..-..A.GCT..AG
